# (E)-Guggulsterone Inhibits Dengue Virus Replication by Upregulating Antiviral Interferon Responses through the Induction of Heme Oxygenase-1 Expression

**DOI:** 10.3390/v13040712

**Published:** 2021-04-20

**Authors:** Wei-Chun Chen, Chih-Ku Wei, Monir Hossen, Yao-Chin Hsu, Jin-Ching Lee

**Affiliations:** 1 Department of Biotechnology and PhD Program in Life Sciences, College of Life Science, Kaohsiung Medical University, Kaohsiung 80708, Taiwan; daphny3016@hotmail.com (W.-C.C.); kweyyang9202@gmail.com (C.-K.W.); 2 Graduate Institute of Medicine in College of Medicine, Kaohsiung Medical University, Kaohsiung 80708, Taiwan; monir.mbg@gmail.com; 3 Chinese Medical Department, Chi-Mei Medical Center, Tainan 71004, Taiwan; 4 Department of Marine Biotechnology and Resources, National Sun Yat-sen University, Kaohsiung 80424, Taiwan; 5 Graduate Institute of Natural Products in College of Pharmacy and Drug Development and Value Creation Research Center, Kaohsiung Medical University, Kaohsiung 80708, Taiwan; 6 Department of Medical Research, Kaohsiung Medical University Hospital, Kaohsiung 80708, Taiwan

**Keywords:** (E)-guggulsterone, DENV, heme oxygenase-1 (HO-1), interferon

## Abstract

Dengue virus (DENV) infection, which causes dengue fever, dengue hemorrhagic fever, and dengue shock syndrome, is a severe global health problem in tropical and subtropical areas. There is no effective vaccine or drug against DENV infection. Thus, the development of anti-DENV agents is imperative. This study aimed to assess the anti-DENV activity of (E)-guggulsterone using a DENV infectious system. A specific inhibitor targeting signal molecules was used to evaluate the molecular mechanisms of action. Western blotting and qRT-PCR were used to determine DENV protein expression and RNA replication, respectively. Finally, an ICR suckling mouse model was used to examine the anti-DENV activity of (E)-guggulsterone in vivo. A dose-dependent inhibitory effect of (E)-guggulsterone on DENV protein synthesis and RNA replication without cytotoxicity was observed. The mechanistic studied revealed that (E)-guggulsterone stimulates Nrf2-mediated heme oxygenase-1 (HO-1) expression, which increases the antiviral interferon responses and downstream antiviral gene expression by blocking DENV NS2B/3B protease activity. Moreover, (E)-guggulsterone protected ICR suckling mice from life-threatening DENV infection. These results suggest that (E)-guggulsterone can be a potential supplement for controlling DENV replication.

## 1. Introduction

Dengue infection is a severe arthropod-borne disease caused by the dengue virus (DENV) and is particularly prevalent in tropical and subtropical areas [1]. More than 3 billion people in 128 countries are currently at risk of dengue infection, and almost 400 million DENV infections occur globally each year [2]. The consequences of DENV infection range from self-limiting dengue fever (DF) to life-threatening dengue hemorrhagic fever (DHF) and dengue shock syndrome (DSS) [3]. Regrettably, there is no FDA-approved anti-dengue viral treatment or a successful vaccination strategy [4].

DENV is a positive-stranded RNA virus, is enclosed by an enveloped protein, and a member of the Flaviviridae family and Flavivirus genus. The genome size is about 11 kb, encoding three structural proteins (capsid protein C, membrane protein prM, and envelope protein E) and seven nonstructural proteins (NS1, NS2A, NS2B, NS3, NS4A, NS4B, and NS5) [5]. Among these, NS2B/NS3 protease supports viral genome replication into the host by hindering the cellular type I interferon (IFN) signaling pathway through interaction with cellular IκB kinase ε (IKKε) and cleavage of human mediator of IRF3 activation (MITA) [6,7].

Similar to HIV and HCV, DENV infection also triggers oxidative stress by the NADPH oxidase in DENV-infected cells. In addition, DENV-induced oxidative stress enhances inflammatory cytokine secretion, which is correlated with DENV pathogenesis [8,9]. In the feedback responses, several antioxidant enzymes such as heme oxygenase-1 (HO-1), superoxidase dismutase (SOD-2), and glutamate-cysteine ligase (GCLM) are activated through the Nrf2 (Nuclear factor erythroid 2-related factor 2)/Keap1 (kelch-like ECH-associated protein 1) pathway to quench DENV-induced oxidative stress as well as suppress DENV replication [10,11]. The HO-1 antioxidant enzyme breaks down the heme ring into biliverdin, carbon monoxide, and ferrous iron that act as cryoprotectants [12]. Based on previous studies, the anti-DENV effect of HO-1 is mainly exerted by biliverdin, which activates the host antiviral IFN responses by non-competitively inhibiting DENV NS2B/NS3 protease activity [11,13,14]. In addition, DENV NS2B/NS3 can also manipulate Nrf2-mediated antioxidant network system by degrading Nrf2 protein at the later times of infection (>24 hpi). Furthermore, degradation of Nrf2 with DENV NS2B/NS3 leads to increased oxidative stress and facilitates DENV replication [15]. These phenomena suggest that induction of HO-1 enzyme activity will be an effective way to reduce DENV-infected oxidative as well as to control DENV replication.

Guggulsterone, a well-known ayurvedic supplement derived from *Commiphora mukul*, has a vast range of therapeutic applications, including arthritis, inflammation, cardiovascular disease, diabetes, and hyperlipidemia [16]. A previous study described that (E)-guggulsterone, natural stereoisomers of guggulsterone, markedly upregulate HO-1 expression via Nrf2 activation [17]. Additionally, (E)-guggulsterone displays virucidal activity against HSV-2, CVB-3, and RSV-B, but anti-DENV efficacy has not been reported thus far [18]. In this study, we demonstrated that (E)-guggulsterone suppresses DENV replication by upregulating antiviral interferon responses by inducing heme oxygenase-1 expression. Similarly, (E)-guggulsterone also shielded ICR suckling mice from life-threatening DENV infection. Our findings suggested that (E)-guggulsterone is a possible candidate for the treatment of DENV infection.

## 2. Materials and Methods

### 2.1. Ethics Statement and Experimental Animals

Six-day-old ICR suckling mice were used in this study; the ICR breeder mice were obtained from BioLasco, Taipei, Taiwan Co. Ltd. All animal experiments were performed in specific pathogen-free (SPF) environments in compliance with the guidelines of the Care and Use of Laboratory Animals. The experimental protocol was approved by the Animal Research Committee of Kaohsiung Medical University of Taiwan (IACUC, 106162) under the guidance of the Public Health Service (PHS) Policy on Humane Care and Use of Laboratory Animals. All mice received humane care and were fed with standard rodent chow and water ad libitum. The mice were acclimated under standard laboratory conditions following the Animal Use Protocol of Kaohsiung Medical University for 1 week before the experiment.

### 2.2. Cell Culture, Virus, and Reagents

Huh-7 cells were cultured in Dulbecco’s modified Eagle’s medium (DMEM) containing 10% fetal bovine serum, 1% non-essential amino acids, and 1% antibiotic-antimycotic with 5% CO2 at 37 °C. DENV (type 2 strain PL046 and 16681) was propagated in C6/36 mosquito cells according to a prior laboratory protocol [19]. (E)-Guggulsterone (CAS number 29025-24-6) and tin mesoporphyrin IX (SnMP) (CAS number 553-12-8) were purchased from Sigma (Sigma, St. Louis, MO, USA). Vero.STAT1 KO cell line, a STAT1 gene knockout Vero cells, was purchased from American Type Culture Collection (ATCC, Manassas, WV, USA; ATCC number CCL-81-VHG^TM^). SnMP, a porphyrin derivative, is a competitive inhibitor of heme oxygenase. Besides, due to its enzyme activity inhibition, it can block bilirubin production instead of affecting heme oxygenase expression. All chemicals are solved in DMSO and diluted by fresh medium before treatment.

### 2.3. DENV Infection in Huh-7 Cells

Overnight seeded Huh-7 cells (5 × 10^4^/well) in 24-well plates were infected with DENV at an MOI of 0.2. After infection for 2 hours, the viral suspension was removed, and the cells were treated with various concentrations of (E)-guggulsterone for 3 days. The DENV protein and RNA were then analyzed by Western blotting and RT-qPCR, respectively.

### 2.4. Western Blotting

The cells were lysed using RIPA lysis buffer, and the lysates were collected for Western blotting assay. In brief, equal loading volumes of cell lysates were analyzed by SDS-PAGE, followed by transfer to a PVDF membrane. The membrane samples were probed with antibodies specific for anti-DENV NS2B antibody (1:3000; GeneTex, GTX124246, Inc, Irvine, CA, USA), anti-GAPDH antibody (1:10,000; GeneTex, GTX112827), anti-Nrf2 antibody (1:3000; GeneTex, GTX103322), anti-Lamin B1 (GTX103292), anti-Tubulin (GTX112141), anti-Histone H1 (GTX87506) antibody (1:10,000; GeneTex), and anti-HO-1 antibody (1:3000; Abcam ab13243, Cambridge, MA, USA). The protein abundance of the samples was quantified using ImageJ software following densitometric scanning [20].

### 2.5. DENV and Cellular mRNA Quantification

Total cell RNA was extracted using an RNA extraction kit (GeneMark Biolab Co., Ltd., Taipei, Taiwan) following the manufacturer’s instructions. DENV RNA and cellular mRNAs levels were analyzed by quantitative real-time reverse-transcription polymerase chain reaction (RT-qPCR) as previously described [11]. The gene expression levels were normalized using cellular glyceraldehyde-3-phosphate dehydrogenase (gapdh) mRNA. The primers used in the study are listed in Table 1.

### 2.6. Cytotoxicity Assay

Huh-7 cells were seeded in 96-well plates at 5 × 10^3^ cells per well and treated with (E)-guggulsterone at the indicated concentrations. The cell viability was assessed using the CellTiter 96 Aqueous One Solution Cell Proliferation Assay (Promega, Madison, WI, USA) according to the manufacturer’s instructions. The color intensity was detected at 490 nm using a 550 BioRad plate-reader (Biorad, Hertfordshire, UK).

### 2.7. Transfection and Luciferase Activity Assay

Huh-7 cells were seeded in 24-well plates at 5 × 10^4^ per well and transfected with specific plasmids (including pHO-1-Luc, p3×ARE-Luc, pISRE-Luc) using the T-Pro™ transfection reagent according to the manufacturer’s instructions. The transfected cells were treated with the indicated (E)-guggulsterone for 3 days. Luciferase activity was analyzed using the Steady-Glo Luciferase Assay System (Promega, Madison, WI, USA) as previously described [13]. Here, pHO-1-Luc, containing a human HO-1 promoter driving firefly luciferase expression, was used to measure the HO-1 transcriptional activity. p3×ARE-Luc harboring three repeats of the Nrf2-dependent antioxidant response element (ARE) was used to detect the translocation and transcription activity of Nrf2. pISRE-Luc, a reporter vector containing firefly luciferase under the control of an IFN-stimulated response element (ISRE), was used to measure IFN response-dependent transcriptional activity.

### 2.8. DENV NS2B/NS3 Protease Assay

The DENV NS2B/NS3 protease assay was performed as previously described [11]. In brief, Huh-7 cells were co-transfected with 0.25 μg of NS2B/NS3 protease reporter vector pEG(∆4B/5)NLuc and 0.75 μg of non-autocleavage NS2B/NS3 protease vector pNS2B(G_4_SG_4_)NS3 and then incubated with (E)-guggulsterone at concentrations of 2.5, 5, 10, or 20 μM for 3 days. Transfection of the dysfunctional NS2B/NS3 protease expression vector, pNS2B (G_4_SG_4_) NS3^mut^, carrying double-point mutations (L75A/S135A) in the protease activity site, served as a negative control. For evaluating the trans-acting activity of the DENV NS2B/NS3 protease in the DENV infection system, DENV-infected Huh-7 cells were transfected with 1 μg of NS2B/NS3 protease reporter vector pEG (∆4B/5) NLuc, and then the cells were then treated with (E)-guggulsterone at increasing concentrations for 3 days. After 3 days, the supernatants were harvested for the nano luciferase activity assay using the Nano-Glo^®^ Luciferase Assay System (Promega). Each transfection mixture contained 0.1 μg of the firefly luciferase expression vector (pCMV-FLuc) as a transfection efficacy control for normalization against the nano-luciferase activity.

### 2.9. Analysis of Extracellular IFN-α Protein Level

Huh-7 cells were seeded in 24-well plates and then infected with DENV at an MOI of 0.2 for 2 hours, followed by (E)-guggulsterone treatment. After 3 days, the supernatants were collected, and the IFN-α concentrations were measured using a human IFN-α ELISA kit (Invitrogen, Thermo Fisher Scientific Inc., Waltham, MA, USA) according to the manufacturer’s protocol. The absorbance was detected at 450 nm using an Epoch microplate spectrophotometer (BioTek Instruments Inc., Winooski, VT, USA).

### 2.10. Anti-DENV Activity Assay In Vivo

Six-day-old ICR suckling mice were randomly divided into four groups (five mice each group); Group 1 received 60 °C heat-inactivated DENV and saline treatment (iDENV); Group 2 received 2.5 × 10^5^ plaque-forming unit (PFU) of DENV and saline treatment (DENV); and Group 3 received 2.5 × 10^5^ PFU of DENV and 5 mg/kg of (E)-guggulsterone treatment at 1, 3, and 5 days post-infection (DENV + (E)-guggulsterone 5 mg/kg). Group 4 received 2.5 × 10^5^ PFU of DENV and 10 mg/kg of (E)-guggulsterone treatment at 1, 3, and 5 days post-infection (DENV + (E)-guggulsterone 10 mg/kg). The body weights and mortality rates of the mice in each group were recorded daily for up to 6 days. For determination of the viral titer, 6-day-old ICR suckling mice were randomly divided into two groups (five mice, each group); Group 1 received 2.5 × 10^5^ PFU of DENV and saline treatment (DENV); Group 2 received 2.5 × 10^5^ PFU of DENV and 10 mg/kg of (E)-guggulsterone treatment at 1, 3, and, 5 days post-infection (DENV + (E)-guggulsterone 10 mg/kg). The suckling mice were sacrificed after 6 days by carbon dioxide euthanasia. The brain tissues were collected, weighed, and homogenized in 0.5 mL RPMI 1640 medium supplemented with 2% FBS and then centrifuged at 8000 rpm for 15 min at 4 °C. Finally, a plaque assay was performed according to our laboratory protocols [11].

### 2.11. Statistical Analysis

The data are expressed as mean ± SD of at least three independent experiments. Statistical analysis was accomplished using the Student’s t-test; *p*-values < 0.01 were considered statistically significant.

## 3. Results

### 3.1. (E)-Guggulsterone Inhibits DENV Protein Synthesis and RNA Replication

**Figure 1:**
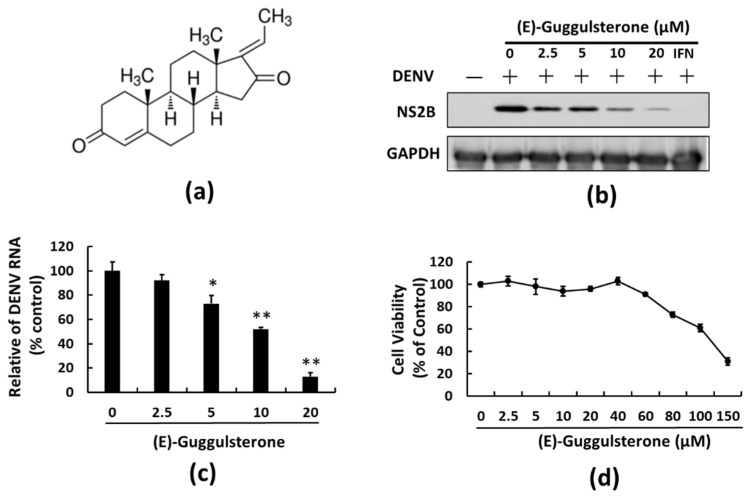
(E)-Guggulsterone inhibits DENV replication in a dose-dependent manner. (**a**) (E)-guggulsterone chemical structure. (E)-guggulsterone efficiently inhibited DENV (**b**) protein synthesis and (**c**) RNA replication in a dose-dependent manner. DENV-infected Huh-7 cells were treated with 0.1% of DMSO (dose 0, consider as the negative control), 2.5, 10, or 20 μM (E)-guggulsterone for 3 days. The DENV protein and RNA levels were analyzed by Western blotting and RT-qPCR, respectively. The percent inhibition was relative to the cells treated with 0.1% DMSO (corrected to “0”) with DENV infection defined as 100%. (**d**) Cell viability of (E)-guggulsterone. Huh-7 cells were treated with the indicated concentrations of (E)-guggulsterone for 3 days, and the cell viability was assessed using an MTS assay. Error bars denote the mean ± SD of three independent experiments (*n* = 3). * *p* < 0.05; ** *p* < 0.01.

(E)-guggulsterone (Figure 1a) is a general isoform of guggulsterone having antiviral efficacy against four distinct viruses [18]. We treated DENV-infected Huh-7 cells with the indicated concentrations of (E)-guggulsterone for 3 days to assess the anti-DENV properties of (E)-guggulsterone. DENV protein and RNA levels were then analyzed by Western blotting and RT-qPCR with specific antibodies and primers, respectively. The result showed that (E)-guggulsterone treatment reduced DENV protein synthesis (Figure 1b) and RNA levels (Figure 1c) with an EC_50_ value of 8.7 ± 1.5 μM. Moreover, assessment of cell viability using an MTS assay indicated no cytotoxic effects at an effective concentration of 20 μM (Figure 1d). This suggests that (E)-guggulsterone suppresses DENV replication in a dose-dependent manner without causing cytotoxicity.

### 3.2. (E)-Guggulsterone Suppresses DENV Replication by Inducing HO-1 Expression

**Figure 2:**
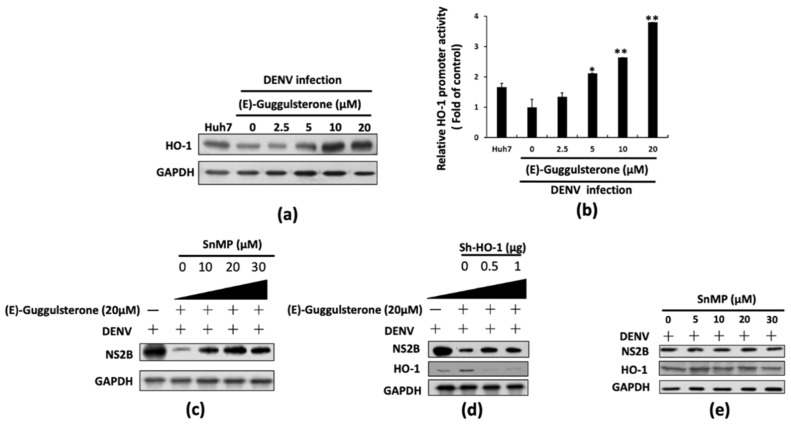
(E)-Guggulsterone inhibits DENV replication through an HO-1 dependent pathway. (**a**) (E)-guggulsterone enhanced HO-1 protein expression in the DENV-infected cells. DENV-infected Huh-7 cells were treated with the indicated concentrations of (E)-guggulsterone for 3 days, and the HO-1 protein levels were analyzed by Western blotting. (**b**) (E)-guggulsterone induced the promoter activity. The HO-1 promoter-reporter plasmid, pHO-1-Luc, was transfected into Huh-7 cells. The plasmid-transfected cells were infected with DENV and treated with the indicated concentrations of (E)-guggulsterone (0–20 μM) for 3 days. The cell lysates were then subjected to luciferase activity assay. The relative luciferase activity is presented as fold activation compared to 0.1% DMSO-treated cells (designated as “0”), with DENV infection, defined as “1”. (**c**,**d**) Inhibition of HO-1 activity or silencing of HO-1 expression attenuated the inhibitory effect of (E)-guggulsterone on DENV replication. DENV-infected Huh-7 cells were treated with different concentrations of HO-1 inhibitor SnMP (10, 20, 30 μM) or co-transfected with different concentrations of plasmid encoding HO-1 shRNA, pCMV-sh-HO-1, (0.5 and 1 μg) in the absence or presence of (E)-guggulsterone (20 μM) for 3 days. (**e**) SnMP shows no significant effect on DENV replication and HO-1 expression. DENV-infected Huh-7 cells were treated with different concentrations of SnMP (10, 20, and 30 μM) for 3 days. The DENV and HO-1 protein synthesis was assessed using Western blotting. The treatment with 0.1% DMSO was defined as “0”. The error bars for treatment denote the mean ± SD of three independent experiments (*n* = 3). * *p* < 0.05; ** *p* < 0.01.

According to our previous studies, an HO-1 inducer effectively suppressed DENV replication [11,13], and other research has indicated that (E)-guggulsterone also can induce The HO-1 expression in the human mammary epithelial cells [17]. We first performed Western blotting to analyze the HO-1 protein expression levels in response to (E)-guggulsterone treatment to determine whether (E)-guggulsterone stimulates HO-1 expression in DENV-infected Huh-7 cells to block DENV replication. As shown in Figure 2a, (E)-guggulsterone dose-dependently increased HO-1 protein expression. Furthermore, we performed an HO-1 promoter activity assay with the HO-1 promoter-driven firefly luciferase expression vector pHO-1-Luc to examine the transcriptional activation by (E)-guggulsterone. Similarly, (E)-guggulsterone also dose-dependently increased DENV-suppressed HO-1 promoter activity (Figure 2b). We further designed a restoration experiment for DENV replication by attenuating HO-1 expression or activity using specific HO-1 inhibitor SnMP in the presence of (E)-guggulsterone to verify that the anti-DENV activity of (E)-guggulsterone was due to HO-1 activation. Huh-7 cells were treated with (E)-guggulsterone alone or co-treated with (E)-guggulsterone and SnMP for 3 days. Western blotting demonstrated that DENV protein levels in the (E)-guggulsterone-treated cells were gradually restored in the presence of SnMP (Figure 2c). We also observed that the anti-DENV activity of (E)-guggulsterone was gradually attenuated by knockdown of HO-1 expression (Figure 2d). As a control, there is no significant effect on DENV replication and HO-1 expression upon SnMP treatment in the DENV-infected Huh-7 cells (Figure 2e). These observations, taken together, confirm our assertion that the upregulation of HO-1 expression by (E)-guggulsterone leads to antiviral control.

### 3.3. (E)-Guggulsterone Upregulates Nrf2 for HO-1 Expression

**Figure 3:**
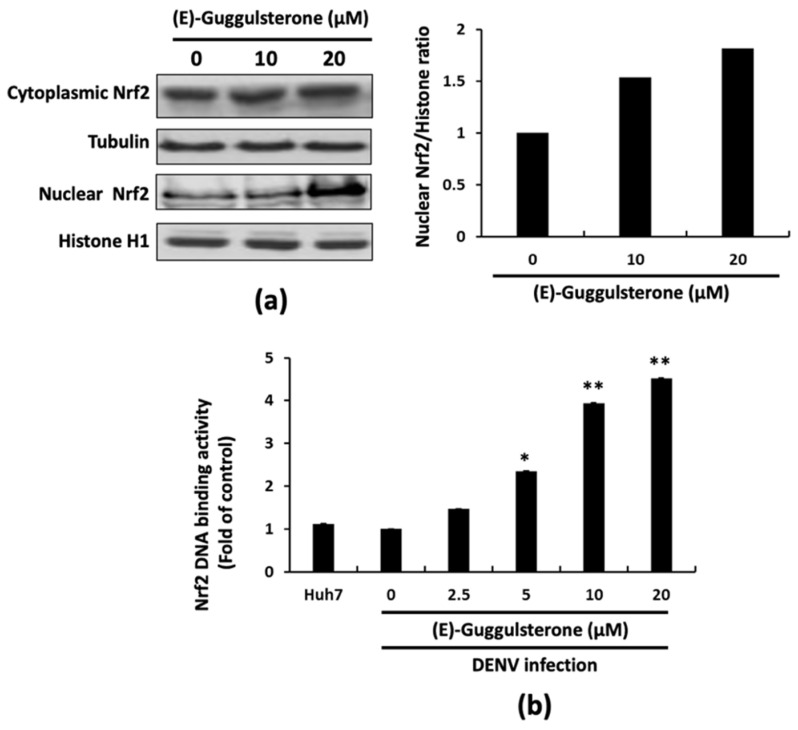
(E)-Guggulsterone upregulates Nrf2 for HO-1 expression. (E)-Guggulsterone enhances cytoplasmic Nrf2 expression and Nrf2 nuclear translocation. (**a**) DENV-infected cells were treated with two different concentrations of (E)-guggulsterone (10 and 20 μM). The cytoplasmic and nuclear lysates were extracted separately, and Western blotting was performed using anti-Nrf2, anti-Tubulin, and anti-Histone H1 antibodies. Protein concentrations were quantified using densitometric analysis. (**b**) The (E)-guggulsterone stimulated ARE transactivation in a concentration-dependent manner. Huh-7 cells were transfected with the reporter plasmid p3xARE-Luc and infected with DENV for 2 h, followed by treatment with the indicated concentration of (E)-guggulsterone for 3 days. The relative induction of promoter activity was determined using a luciferase assay. The results are presented as fold activation compared to the DENV-infected cells, with 0.1% DMSO treatment defined as “0”. The error bars for treatment denote the mean ± SD of three independent experiments (*n* = 3). * *p* < 0.05; ** *p* < 0.01.

Three major transcription factors, Nrf2, Keap1, and Bach1, are associated with HO-1 expression [21], and among them, Nrf2 suppresses DENV replication via enhancement of HO-1 expression, as demonstrated by our previous study [13]. We first analyzed the effects of (E)-guggulsterone on the protein expression of Nrf2 factors in DENV-infected cells to prove (E)-guggulsterone also activates HO-1 expression by translocating the Nrf2 nuclear factor. Western blot results bolstered the finding that (E)-guggulsterone increases the overall protein levels of Nrf2 and the nuclear aggregation of Nrf2 in a concentration-dependent manner (Figure 3a). Furthermore, we used a p3xARE-Luc reporter vector containing three tandem copies of the Nrf2-dependent antioxidant response element (ARE) linked to luciferase to measure the transcriptional activity of nuclear Nrf2. The reporter-transfected cells were infected with DENV and treated for 3 days with increasing (E)-guggulsterone concentrations. (E)-guggulsterone massively enhanced luciferase activity in a concentration-dependent manner, indicating that (E)-guggulsterone activated the Nrf2 transcription factor for induction of HO-1 expression (Figure 3b). These findings together demonstrate that the antiviral activity of (E)-guggulsterone is based on HO-1 stimulation activated by the Nrf2 transcription factor.

### 3.4. (E)-Guggulsterone Remarkably Inhibits DENV NS2B/NS3 Protease Activity

**Figure 4:**
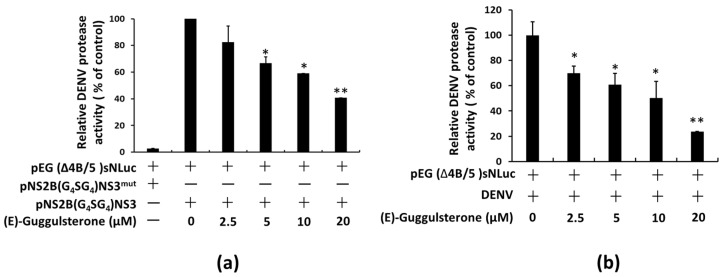
(E)-Guggulsterone inhibits DENV NS2B/NS3 protease activity. (**a**) Huh-7 cells were co-transfected with the protease reporter vector, pEG (Δ4B/5) sNLuc, and the protease expression vector, pNS2B (G4SG4) NS3, or the mutant protease expression vector, pNS2B (G4SG4) NS3mut. (**b**) DENV infected Huh-7 cells were transfected with the protease reporter vector. The plasmid-transfected cells were treated with (E)-guggulsterone at the indicated concentrations for 3 days. DENV NS2B/NS3 protease activity was measured by detecting the secreted nano-luciferase activity in the supernatant. Each transfection mixture contained 0.1 μg of the firefly luciferase expression vector as a transfection control for normalization against the nano-luciferase activity. The relative percent inhibition was compared to protease-expressing or DENV-infected cells, defined as 100%. The results are expressed as the mean ± SD (error bar) of three independent experiments (*n* = 3); * *p* < 0.05, ** *p* < 0.01.

According to our previous study, lucidone, a natural product with strong HO-1 inducing activity, minimizes DENV NS2B/NS3 protease activity by HO-1 metabolite biliverdin and stimulates antiviral IFN responses in vitro and in vivo [11,13]. Here, Huh-7 cells were co-transfected with pEG (Δ4B/5) sNLuc reporter vector and the DENV NS2B/NS3 protease expression vector pNS2B (G_4_SG_4_) NS3, for which co-transfection with a mutant protease pNS2B (G_4_SG_4_) NS3^mut^ served as a negative control, to investigate the inhibitory effect of (E)-guggulsterone on DENV NS2B/NS3 protease activity. The plasmid-transfected cells were treated with various concentrations of (E)-guggulsterone for 3 days, then the luciferase assay was used to measure the DENV NS2B/NS3 protease activity. (E)-guggulsterone dose-dependently decreased DENV NS2B/NS3 protease activity (Figure 4a). Similarly, (E)-guggulsterone also dose-dependently reduced the DENV NS2B/NS3 protease activity under DENV infection as determined with a luciferase activity assay (Figure 4b).

### 3.5. (E)-Guggulsterone Induces Interferon-α Expression AGAINST DENV Infection

**Figure 5:**
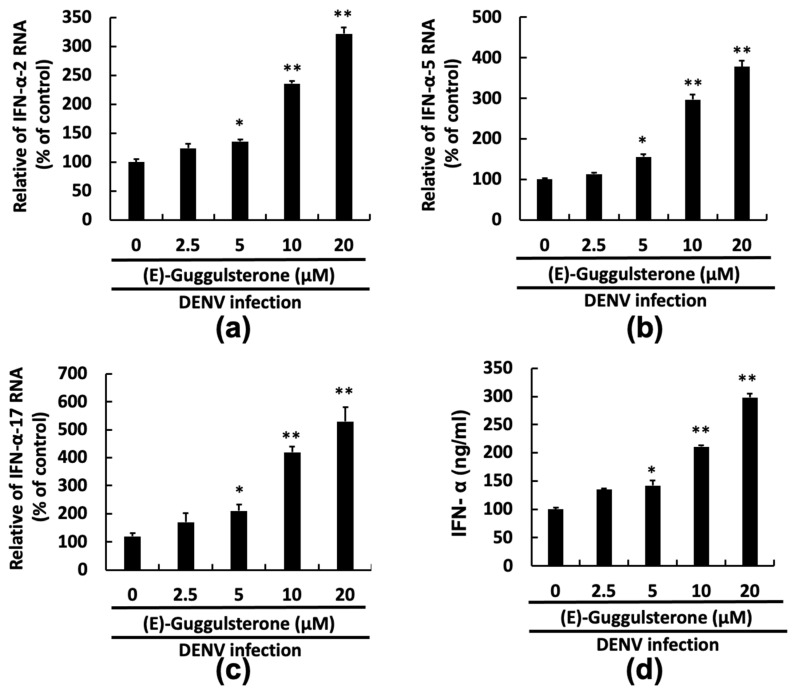
(E)-Guggulsterone induces antiviral IFN responses in DENV-infected Huh-7 cells. (E)-Guggulsterone increased the mRNA levels of (**a**) IFN-α-2, (**b**) IFN-α-5, and (**c**) IFN-α-17 in DENV-infected Huh-7 cells. Huh-7 cells were infected with DENV and then treated with the indicated concentrations of (E)-guggulsterone (0–20 μM) treatment for 3 days. The mRNA level of IFN-α was analyzed using RT-qPCR. (**d**) (E)-guggulsterone upregulated DENV-suppressed IFN-α protein expression in DENV infected Huh-7 cells. ELISA was used to determine the IFN-α protein levels in the culture media. The relative percent induction was compared to the DENV-infected cells treated with 0.1% DMSO, defined as 100%. Here “0” was regarded as the treatment with 0.1% DMSO. Error bars denote the mean ± SD of three independent experiments (*n* = 3); * *p* < 0.05, ** *p* < 0.01.

Studies have conveyed that DENV infection interferes with the antiviral IFN response, as caused by the DENV NS2B/NS3 protease [7,22]. However, according to our prior reports, DENV NS2B/NS3 protease inhibitors increased the DENV-reduced IFN signal, reflecting the antiviral action [11,13]. Here, we first infected cells with DENV and treated them with various (E)-guggulsterone concentrations for 3 days and then measured the expression of IFN-α-2, IFN-α-5, and IFN-α-17 genes by using qRT-PCR. Since DENV reduces IFN gene expression to escape innate immunity, we observed that the (E)-guggulsterone significantly enhances the mRNA expression of IFN-related genes, including IFN-α-2, IFN-α-5, and IFN-α-17, in a dose-dependent manner (Figure 5a–c). The IFN-α protein secreted in the supernatant was also detected by ELISA, showing that (E)-guggulsterone stimulated DENV-reduced IFN-α levels in a concentration-dependent manner (Figure 5d), suggesting that (E)-guggulsterone induces antiviral IFN responses against DENV infection.

### 3.6. (E)-Guggulsterone Activates IFN-Mediated Antiviral Responses against DENV Infection

**Figure 6:**
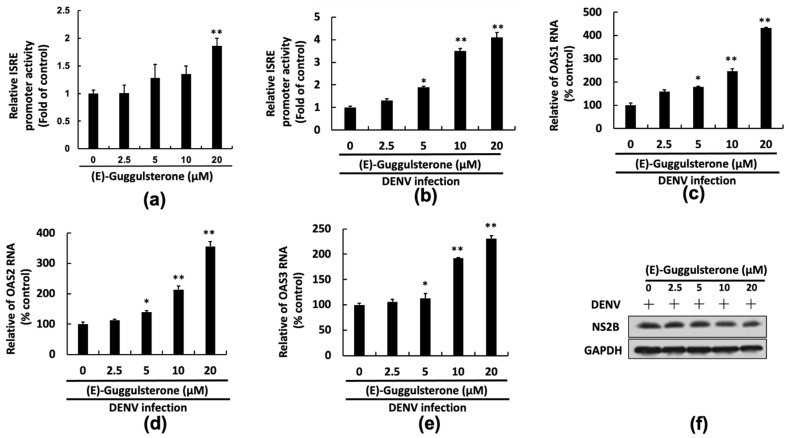
(E)-Guggulsterone induces IFN-mediated antiviral gene expression. (**a**,**b**) (E)-Guggulsterone induced the ISRE promoter activity in parental Huh-7 and DENV-infected Huh-7 cells. Huh-7 cells were transfected with pISR-Luc and then treated with the indicated concentrations of guggulsterone (0–20 μM) for 3 days. The relative induction of ISRE promoter activity was determined using a luciferase assay. The relative induction of promoter activity was determined by luciferase assay and presented as fold activation compared to the DENV-infected cells treated with 0.1% DMSO, designed as “0”. (E)-Guggulsterone increased the mRNA levels of (**c**) OAS1, (**d**) OAS2, and (**e**) OAS3 in DENV-infected Huh-7 cells. DENV-infected Huh-7 cells were treated with the indicated (E)-guggulsterone concentrations (0–20 μM) for 3 days. The mRNA level of OAS1-3 was analyzed using RT-qPCR. The relative percent induction was compared to the DENV-infected cells treated with 0.1% DMSO, defined as 100%. (**f**) (E)-guggulsterone exhibited slightly antiviral activity in Vero.STAT1 KO cells. DENV-infected Vero.STAT1 KO cells were treated with the indicated concentrations of (E)-guggulsterone for 3 days, and the DENV protein levels were analyzed by Western blotting. Here, “0” was regarded as the treatment with 0.1% DMSO. Error bars indicate the mean ± SD of five independent experiments (*n* = 5). * *p* < 0.05, ** *p* < 0.01.

The IFN signal activates the interferon-stimulated response element (ISRE) promoter region, leading to transcription of several anti-DENV genes, of which encoding 2′-5′-oligoadenylate synthetase (OAS) is notable [23]. We transfected pISRE-Luc reporter plasmid into Huh-7 cells and then infected cells with or without DENV, followed by treatment with (E)-guggulsterone for 3 days to verify that (E)-guggulsterone increased the ISRE promoter activity. (E)-guggulsterone augmented the ISRE promoter activity, as indicated by luciferase activity, in the presence or absence of DENV infection (Figure 6a,b). We further examined the effect of (E)-guggulsterone on the transcription response of the OAS1, OAS2, and OAS3 genes during DENV infection. The results indicated that (E)-guggulsterone treatment enhanced the gene expression of the OAS1, OAS2, and OAS3 (Figure 6c–e). To further identify the IFN-dependent antiviral activity of (E)-guggulsterone, we use a STAT1 knockout Vero cell line that lacks an IFN-based antiviral response to investigate the anti-DENV effect of (E)-guggulsterone. The result of Western blotting showed that (E)-guggulsterone exhibited slightly antiviral activity in the IFN-deficient cells (Figure 6f). Based on these in vitro studies, (E)-guggulsterone hinders DENV replication by stimulating Nrf2-mediated HO-1 expression, which increases the antiviral IFN responses and downstream antiviral gene expression by targeting DENV NS2B/3B protease activity.

### 3.7. (E)-Guggulsterone Decreases Mortality in DENV Infected ICR Suckling Mice

**Figure 7:**
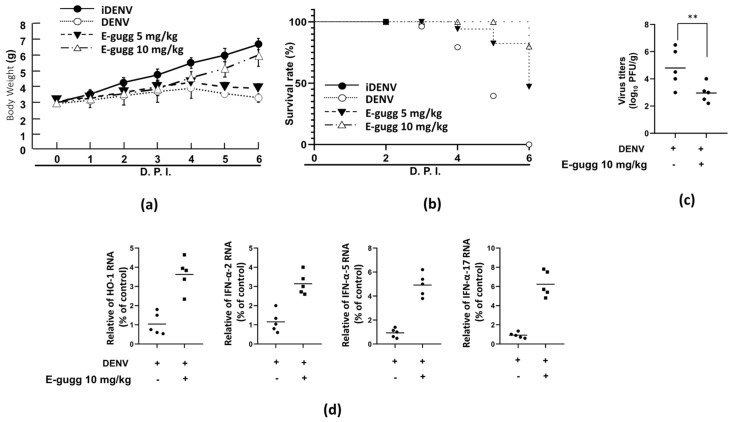
(E)-Guggulsterone delays DENV-2-induced lethality in the suckling mouse model. Six-day-old ICR suckling mice were injected intraperitoneally with 2.5 × 10^4^ pfu DENV or heat-inactivated DENV (iDENV) (control group). DENV-infected mice were administrated either (E)-guggulsterone (5 and 10 mg/kg), marked as E-gugg, or saline (negative control) intraperitoneally at 1, 3, and 5 dpi. The (**a**) survival rate and (**b**) body weights of the mice were recorded daily measured up to 6 dpi. The (**c**) DENV titer and (**d**) gene expression of HO-1, IFN-α-2, (**b**) IFN-α-5, and (**c**) IFN-α-17 in the brain tissue were determined at 6 dpi using a plaque assay and RT-qPCR, respectively. Heat-inactivated DENV (iDENV) was administrated to the control group. Each group included 5–10 ten mice. ** *p* < 0.01.

Infectious DENV or heat-inactivated DENV (iDENV) was intraperitoneally injected into 6-day-old ICR suckling mice to confirm the anti-DENV activity of (E)-guggulsterone in vivo. The DENV-infected mice were then treated with either saline or (E)-guggulsterone (5 and 10 mg/kg) at 1, 3, and 5-days post-infection (dpi). The body weights and survival rates of the infected mice with or without (E)-guggulsterone treatment were recorded daily. As shown in Figure 7, the (E)-guggulsterone treatment group gradually recovered body weight compared to the untreated DENV-infected mice by (7a). Notably, (E)-guggulsterone treatment also prolonged the survival rate of DENV-infected mice by 20% and 60% at doses of 5 mg/kg and 10 mg/kg, respectively, compared to the untreated DENV-infected mice at 6 dpi (7b). Treatment with 10 mg/kg (E)-guggulsterone reduced the viral load and activated gene expression of HO-1, IFN-α-2, IFN-α-5, and IFN-α-17 in the DENV-infected mouse brain compared with the untreated group (7c and 7d). Taken together, (E)-guggulsterone therapy conferred a preventive effect against both DENV-induced lethality and also suppressed DENV infection.

## 4. Discussion

**Figure 8:**
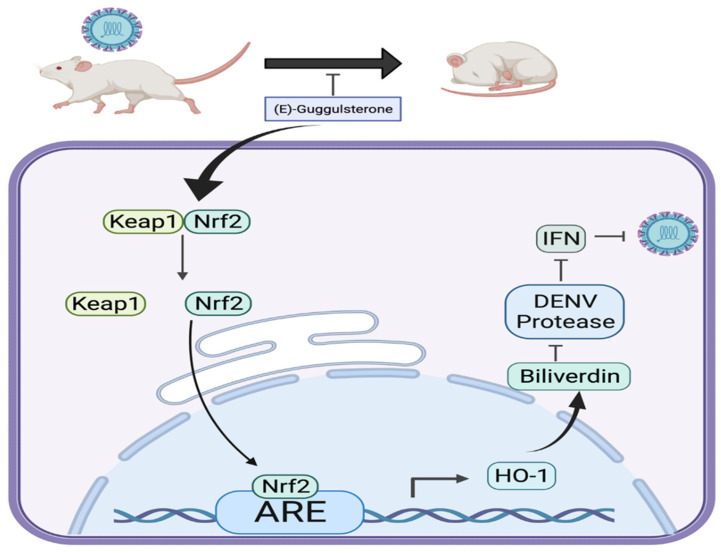
Graphic representation of antiviral action of (E)-guggulsterone in DENV replication. (E)-guggulsterone effectively inhibits DENV replication through the Nrf2/HO-1 signaling pathway, blocks DENV NS2B/NS3 protease activity by stimulating the antiviral IFN response, and delays DENV-induced lethality of ICR suckling mice.

Our results indicate that (E)-guggulsterone effectively hinders the DENV replication (Figure 1) and upregulates DENV-reduced HO-1 expression (Figure 2) in a dose-dependent manner. This finding is consistent with our previous studies showing that the HO-1 inducers lucidone and microRNA-155 inhibit DENV replication [13,24], which is comparable with the results of genetic knockdown by HO-1 shRNA that attenuated the antiviral effects of (E)-guggulsterone (Figure 2d). Another study demonstrated that DENV NS2B/NS3 protease, a vital protein for DENV replication [25], interferes with the antiviral IFN signaling pathway [22]. Here, we found that (E)-guggulsterone can inhibit DENV NS2B/NS3 protease activity (Figure 4) and stimulate the antiviral IFN response in DENV-infected Huh-7 cells (Figure 5 and Figure 6). In addition, the dependence of (E)-guggulsterone antiviral activity was further confirmed by using cells that lack interferon-mediated antiviral response (Figure 6e), which propose that (E)-guggulsterone can induce an antiviral IFN response and act as a selective DENV NS2B/NS3 protease inhibitor. In this study, we determined how (E)-guggulsterone induces HO-1 expression and elucidated its protective mechanisms against DENV infection. DENV triggers oxidative stress associated with viral pathogenesis, and both endogenous and exogenous antioxidants regulate cellular oxidative stress [26]. Similarly, Nrf2 (nuclear factor erythroid 2-related factor 2) is also associated with the regulation of antioxidant gene expression. In brief, DENV infection induces oxidative stress through the effect of NADPH oxidase activity, which activates the inflammatory cytokines response. In the response to oxidative stress, the virus-infected cells rapidly activate Nrf2/Keap1 pathway to mitigate oxidative stress. In normal conditions, Nrf2 and Keap1 form an inactive cytosolic complex, but during oxidative stress, Nrf2 protein disassociates from Keap1 and translocates to the nucleus to promote transcription of antioxidant genes through bind with the ARE region [9,10,14]. Herein, we found that (E)-guggulsterone increased the Nrf2 protein accumulation along with HO-1 expression in the DENV-infected cells (Figure 3), which is in agreement with a prior study that found (E)-guggulsterone induces heme oxygenase-1 expression through activation of Nrf2 in human mammary epithelial cells [17]. According to the results, the present study also demonstrated that treatment with (E)-guggulsterone increased the DENV-infected ICR suckling mice’s HO-1 expression and IFN activation in DENV-infected mice’s brain (Figure 7). Therefore, we can say that (E)-guggulsterone inhibits DENV replication through the Nrf2/HO-1 signaling pathway. The proposed model is illustrated in Figure 8. However, more study is needed to know more precisely how (E)-guggulsterone induces the Nrf2 and HO-1 expression.

(E)-guggulsterone, a chemo-preventive phytochemical compound extracted from the *Commiphora mukul* plants, has been used as an Ayurvedic medicine for over 2500 years to treat various disorders, and its antioxidant content reduces oxidative stress [27,28]. Oxidative stress is an important factor in the progression of DENV infection to dengue hemorrhage and plasma leakage conditions [29]; antioxidant molecules neutralize oxidative stress and also alleviate the DENV pathogenesis symptoms of DHF and DSS [11,30], suggesting that the antioxidant activity of (E)-guggulsterone could be utilized to treat DENV pathogenesis. Numerous studies have revealed that (E)-guggulsterone prevents NF-kappaB-dependent COX-2 expression and PGE_2_ production in vitro [16,31]. Intraperitoneal administration of (E)-guggulsterone (30 mg/kg body weight) decreased the COX-2 protein levels in rats with endotoxin-induced uveitis (EIU) [32]. Our previous research demonstrated that selective COX-2 inhibitors or repressors also suppress the DENV infection in vitro and in vivo [19]. The present study found that treatment with (E)-guggulsterone increased the DENV-infected ICR suckling mice’s body weight, survival rate, and also reduced virus load in infected mice’s brain (Figure 7), suggesting that (E)-guggulsterone treatment could improve the symptoms of DENV infection. We plan to use AG129 mice, a widely used animal model for DENV pathogenesis research, to investigate further the effects of (E)-guggulsterone on DENV-induced pathogenesis.

Clinical studies have demonstrated that (E)-guggulsterone is safe for human research. A previous study identified no side effects when participants with arthritis were given a gum guggul in capsule form (500 mg concentrate) with food [33]. Another clinical study was conducted in the USA, where gum guggul (4.5 g daily) was administrated to 40 hyperlipidemia patients for up to 16 weeks. From these findings, we expect that (E)-guggulsterone could be used in the near future in a clinical study as a potential DENV therapy.

## Figures and Tables

**Table 1:** Oligonucleotide sequences for real-time RT-PCR.

Oligonucleotide Name	Sequence 5′-3′
DENV gene oligonucleotide sequences	
5′ NS5	5′-GGAAACCAAGCTGCCCATCA
3′ NS5	5′-CCTCCACGGATAGAAGTTTA
Human gene oligonucleotide sequences	
5′ GAPDH	5′-GTCTTCACCACCATGGAGAA
3′ GAPDH	5′-ATGGCATGGACTGTGGTCAT
5′ OAS1	5′-CAAGCTTAAGAGCCTCATCC
3′ OAS1	5′-TGGGCTGTGTTGAAATGTGT
5′ OAS2	5′-ACAGCTGAAAGCCTTTTGGA
3′ OAS2	5′-GCATTAAAGGCAGGAAGCAC
5′ OAS3	5′-CACTGACATCCCAGACGATG
3′ OAS3	5′-GATCAGGCTCTTCAGCTTGG
5′ IFN-alpha 2	5′-GCAAGTCAAGCTGCTCTG TG
3′ IFN-alpha 2	5′-GAT GGTTTCAGCCTTTTGGA
5′ IFN-alpha 5	5′-AGTTTGATGGCAACCAGTTC
3′ IFN-alpha 5	5′-TCAGAGGAGTGTCTTCCACT
5′ IFN-alpha 17	5′-AGGAGTTTGATGGCAACCAG
3′ IFN-alpha 17	5′-CATCAGGGGAGTCTCTTC CA

## Data Availability

All data are already presented in the manuscript and available on request from the corresponding author.
